# Aversive cues fail to activate *fos* expression in the asymmetric olfactory-habenula pathway of zebrafish

**DOI:** 10.3389/fncir.2013.00098

**Published:** 2013-05-21

**Authors:** Tagide N. deCarvalho, Courtney M. Akitake, Christine Thisse, Bernard Thisse, Marnie E. Halpern

**Affiliations:** ^1^Department of Embryology, Carnegie Institution for ScienceBaltimore, MD, USA; ^2^Department of Cell Biology, Health Science Center, University of VirginiaCharlottesville, VA, USA

**Keywords:** behavior, asymmetry, alarm pheromone, *fos*, *fam84b*

## Abstract

The dorsal habenular nuclei of the zebrafish epithalamus have become a valuable model for studying the development of left-right (L-R) asymmetry and its function in the vertebrate brain. The bilaterally paired dorsal habenulae exhibit striking differences in size, neuroanatomical organization, and molecular properties. They also display differences in their efferent connections with the interpeduncular nucleus (IPN) and in their afferent input, with a subset of mitral cells distributed on both sides of the olfactory bulb innervating only the right habenula. Previous studies have implicated the dorsal habenulae in modulating fear/anxiety responses in juvenile and adult zebrafish. It has been suggested that the asymmetric olfactory-habenula pathway (OB-Ha), revealed by selective labeling from an *lhx2a:YFP* transgene, mediates fear behaviors elicited by alarm pheromone. Here we show that expression of the *fam84b* gene demarcates a unique region of the right habenula that is the site of innervation by *lhx2a:YFP*-labeled olfactory axons. Upon ablation of the parapineal, which normally promotes left habenular identity; the *fam84b* domain is present in both dorsal habenulae and *lhx2a:YFP*-labeled olfactory bulb neurons form synapses on the left and the right side. To explore the relevance of the asymmetric olfactory projection and how it might influence habenular function, we tested activation of this pathway using odorants known to evoke behaviors. We find that alarm substance or other aversive odors, and attractive cues, activate *fos* expression in subsets of cells in the olfactory bulb but not in the *lhx2a:YFP* expressing population. Moreover, neither alarm pheromone nor chondroitin sulfate elicited *fos* activation in the dorsal habenulae. The results indicate that L-R asymmetry of the epithalamus sets the directionality of olfactory innervation, however, the *lhx2a:YFP* OB-Ha pathway does not appear to mediate fear responses to aversive odorants.

## Introduction

The epithalamus of the teleost brain shows a high degree of left-right (L-R) asymmetry (Concha and Wilson, 2001; Kuan et al., 2007a; Signore et al., 2009); however, the functional significance of this specialization is unknown. In zebrafish, the pineal stalk emerges just to the left of the midline and the accessory parapineal is located to its left (Concha et al., 2000; Liang et al., 2000; Gamse et al., 2002). The adjacent paired habenular nuclei are comprised of dorsal and ventral nuclei, which correspond to the mammalian medial and lateral nuclei, respectively (Amo et al., 2010). The dorsal nuclei can be further divided into asymmetric subnuclei based on their distinct molecular properties (Gamse et al., 2003; Aizawa et al., 2005; Gamse et al., 2005) and the region of the interpeduncular nucleus (IPN) that they innervate (Agetsuma et al., 2010). Thus, the dorsal habenulae display striking differences in size, neuroanatomy, molecular properties, and connectivity.

The asymmetric properties of the zebrafish larval epithalamus arise in a step-wise manner, with Nodal signaling setting the directionality of the pineal complex (Concha et al., 2000; Liang et al., 2000) and the parapineal, which emerges on the left side, providing cues that differentiate the left dorsal habenula from the right (Concha et al., 2003; Gamse et al., 2003; Snelson et al., 2008; Regan et al., 2009). The dorsal habenular nuclei are part of an evolutionarily conserved conduction system between the limbic forebrain and the midbrain via efferent connections to the IPN (Herrick, 1948; Sutherland, 1982; Hikosaka et al., 2008; Bianco and Wilson, 2009). However, in zebrafish, habenular connections to the IPN do not show mirror image symmetry as in mammals (Ramón y Cajal, 1995). Instead, efferents from the left habenula project to the dorsal, intermediate, and ventral regions of the IPN, and the right habenula innervates only the ventral region (Gamse et al., 2005). Further supporting dorsoventral differences at the target, neurons in the dorsal IPN project to the griseum centrale, a region thought to be equivalent to the mammalian periaqueductal gray, whereas those in the ventral IPN innervate the median raphe nucleus (Agetsuma et al., 2010). L-R differences in habenular connectivity thus result in distinct limbic to midbrain pathways on each side of the brain.

In mammals, the habenular region has been implicated in a wide range of behaviors including fear, sex, and feeding (Sutherland, 1982; Bianco and Wilson, 2009; Hikosaka, 2010; Okamoto et al., 2012). The role of the lateral/ventral habenular nuclei in the modulation of emotional behaviors has been intensively studied in rhesus monkey and humans [refer to Hikosaka (2010)]. The function of the medial/dorsal habenula is less well-understood; however, there is evidence that aversive stimuli or stress induce behavioral, biochemical and immunological responses (Silver et al., 1996; Carboni et al., 1998; Cirulli et al., 1998; Sugama et al., 2002; Wilhelm, 2011; Kobayashi et al., 2013).

Recent studies on zebrafish demonstrate that the dorsal habenulae regulate fear-related behaviors. Juveniles with the dorsal habenulae (and other structures) genetically ablated did not learn to avoid an aversive stimulus when paired with a conditional one (Lee et al., 2010). Inactivation of an asymmetric dorsal habenular subnucleus resulted in increased freezing behavior in adult zebrafish in response to a conditioned aversive stimulus (Agetsuma et al., 2010). These studies suggest that the dorsal habenula is an experience-dependent modulator of anxiety and fear-related decision-making (Okamoto et al., 2012).

A known stimulus that elicits fear in fish is “Schreckstoff” or fear substance, an array of chemicals exuded from injured skin, which functions as a predation signal for nearby individuals (Pfeiffer, 1977). Perception of such alarm pheromones in zebrafish provokes rapid darting or freezing, alternative behaviors in response to fear (Waldman, 1982; Speedie and Gerlai, 2008). Because of the dorsal habenula's involvement in modulating fear responses, it has been proposed that alarm pheromone may be the odorant cue for the asymmetrically projecting olfactory-habenula (OB-Ha) pathway (Concha et al., 2012). Furthermore, whole skin extract or glycosaminoglycan chondroitin, results in increased calcium signaling in the medio-dorsal portion of the posterior olfactory bulb (Mathuru et al., 2012), where *lhx2a:YFP* labeled mitral cells are located.

In the present study, we describe a unique domain in the right dorsal habenula characterized by expression of the *fam84b* gene and associated with a discrete neuropil density that corresponds to the site of innervating *lhx2a:YFP* positive olfactory mitral cells. Expression of *fam84b* and the synaptic terminals of the *lhx2a:YFP* neurons are always found in the dorsal habenula that is contralateral to the parapineal. Following removal of the parapineal, *fam84b* expression appears in the left and the right dorsal habenulae and both receive innervation from the olfactory neurons, indicating that directional asymmetry of the epithalamus is sufficient to influence pre-synaptic input. To examine the function of the asymmetric OB-Ha pathway, induction of *fos* expression was used as a measure of neuronal activation in response to a variety of odorants. We find that adult zebrafish exposed to alarm substance extracted from skin, or to the purified component chondroitin sulfate, show robust *fos* activation in the olfactory bulb, but not in the *lhx2a:YFP* subpopulation or in the dorsal habenulae. The results show that the epithalamus directs the formation of an asymmetric telencephalic connection; however, substances that provoke fearful behaviors do not appear to activate this unique OB-HA pathway.

## Materials and methods

### Zebrafish

Zebrafish were housed at 27°C on a 14:10 h light:dark cycle. Zebrafish used in this study were the wild-type AB strain (Walker, 1999) and the transgenic lines *Tg*(*-10lhx2a:GAP-EYFP)*^zf177^ [formerly called *Tg*(*lhx2a*:*gap-YFP*)] and *Tg*(*-10lhx2a:SYP-GFP*)^zf186^ [formerly called *Tg*(*lhx2a*:*Syp-GFP*); Miyasaka et al., 2009] and *Tg*(*foxd3*:GFP)^fkg17^ (Gilmour et al., 2002). To visualize the dorsal habenular nuclei, a transgenic reporter, *TgBAC(gng8:Eco.NfsB-2A-CAAX-GFP)* (DKEY-18313 BAC; Genbank accession number CR450711) that is habenula-specific at 4–5 days post-fertilization (dpf) (Akitake and Halpern, unpublished observations) was also employed. Maintenance of zebrafish and experimental procedures were carried out in accordance with the protocol approved by Institutional Animal Care and Use Committee. Naming of transgenic lines and zebrafish genes (see below) follow the nomenclature guidelines provided by the Zebrafish Model Organism Database (ZFIN).

### RNA *in situ* hybridization and immunofluorescence

The *family with sequence similarity 84, member B* (*fam84b*) gene was identified in an *in situ* hybridization screen of zebrafish cDNAs for their tissue-specific patterns of expression (Thisse and Thisse, 2004). The *fam84b* clone came from a cDNA library prepared from adult zebrafish kidney. Digoxigenin-(DIG) and fluorescein (FITC)-labeled RNA probes were synthesized using reagents from Roche Molecular Biochemical. The *fam84b* plasmid was linearized with NotI and transcribed with SP6 RNA polymerase. A clone of the *v-fos FBJ murine osteosarcoma viral oncogene homolog* gene *(fos*, formerly referred to as *c-fos*) was generated by amplifying a 602 base pair fragment from cDNA prepared from RNA of 3 dpf larvae using forward and reverse primers (TCTCCTCTGTGGCGCCCTCC and GTCTGGAACCGAGCGAGCCG) and subcloning into the pCRII-TOPO vector (Invitrogen). For probe synthesis, the *fos* plasmid was linearized with BamHI and transcribed with T7 RNA polymerase. The *orthodenticle homolog 5* (*otx5*) and *potassium channel tetramerization domain containing 12.1* (*kctd12.1*, formerly called *leftover*) probes were prepared as described (Gamse et al., 2002, 2003). A probe for the *early growth response 1* (*egr1*) gene was synthesized as in Close et al. (2002).

Single and double label colorimetric RNA *in situ* hybridization experiments were performed as previously described for larvae (Gamse et al., 2002) and adult brain tissue (Gorelick et al., 2008). To enhance signal intensity, 5% dextran sulfate (Millipore) was added to the hybridization buffer as in Lauter et al. (2011). Following the colorimetric reaction, adult brains were embedded in 4% low melting point agarose (SeaPlaque, Lonza) and 50 μm coronal sections were collected using a vibratome (Leica VT1000S).

For fluorescent *in situ* hybridization alone or coupled with immunolabeling for yellow fluorescent protein (YFP)/green fluorescent protein (GFP) YFP/GFP, we followed the protocol described in Gamse et al. (2002) until the anti-DIG antibody-blocking step. Samples were placed in maleic acid buffer (MAB) with 2% blocking reagent (Roche) for 1 h according to Lauter et al. (2011). Anti-DIG-antibody conjugated with horseradish peroxidase (Roche) alone or together with anti-GFP rabbit antibody (Torrey Pines TP401) was diluted 1:500 in blocking solution and incubated at 4°C overnight. Probes were visualized by tyramide signal amplification (TSA) reagents that were prepared as described (Vize et al., 2009). Anti-GFP antibody cross-reacts with YFP. Reaction buffer contained 4-iodophenol (Sigma-Aldrich) and 2% dextran sulfate. Tyramide-Cy5 was used for the first *in situ* hybridization probe. For double in *situ* hybridization, reacted samples were incubated in blocking solution for 1 h and then at 4°C overnight in diluted (1:500) anti-FITC-antibody conjugated with horseradish peroxidase (Roche). Tyramide-Cy3 was used for the second *in situ* hybridization probe. Adult brains were embedded in 4% low melting point agarose and 50 μm coronal vibratome sections were collected prior to the TSA detection step. The sections were mounted in Prolong Gold or *Slowfade* Gold (Invitrogen) antifade mounting media. For immunolabeling following fluorescent *in situ* hybridization, reacted samples were placed in blocking solution (0.1% Triton PBS with 10% sheep serum) for 1 h and then in diluted (1:500) goat anti-rabbit-Cy3 antibody (Jackson ImmunoResearch) at 4°C overnight.

### Odor assays

Alarm substance (hereinafter referred to as skin extract) was freshly prepared on the day of testing. Adult fish were immersed in an ice water slurry and decapitated. To prepare skin extract solution, the body was placed in 10 mL distilled water, the skin was abraded with 250 grit sandpaper and 15 shallow cuts were made using a protocol modified from Speedie and Gerlai (2008). Extracts were collected and pooled from 5 male and 5 female individuals (100 mL total) and filtered under vacuum using a 0.2 μm filter (Nalgene).

Adult zebrafish (3–6 months) of both sexes were placed in 1 L breeding tanks (Aquatic Habitats) containing system water and acclimated for 1 h prior to odorant exposure. All experiments were performed with adults because larval zebrafish do not respond to alarm pheromone (Waldman, 1982). For alarm pheromone assays, single fish were placed in tanks separated by black paper. Solutions were delivered via a 5/32 × 9/32 mm diameter tygon tube (Pennplex) entering below the water line. A 10 mL syringe filled with 2.5 mL of the skin extract or chondroitin sulfate solution was connected to the other end of the plastic tubing and the contents slowly injected into each tank for a final concentration of 0.2% skin extract or 1 μg/ml shark chondroitin sulfate (Sigma-Aldrich). Other ecologically and behaviorally relevant odors were tested in a similar manner; however, groups of 5 fish were placed in one tank for each odorant. Test chemicals were purchased through Sigma-Aldrich and used at a final concentration of 400 μM for L-cysteine and putrescine and 40 μM for cadaverine and trimethylamine. Ground fish flakes (100 mg/L; Tetramin Tropical Flakes), 0.1% bile obtained from freshly killed tilapia (*Orechromis mossambicus*) and 0.25% of a conspecific extract, which was obtained by grinding and straining 10 freshly sacrificed adult zebrafish of both sexes into a 10 ml of distilled water were also tested. Control individuals received only distilled water. Following odorant exposure, fish were held in the experimental tanks for 30 min, then immersed in an ice water slurry and decapitated. Brains were immediately dissected in cold PBS and fixed in 4% paraformaldehyde at 4°C overnight.

As a positive control, the glutamate receptor agonist, kainic acid, was used to elicit *fos* expression in *lhx2a:YFP* labeled mitral cells. Primary sensory neurons excite mitral cells through AMPA and kainic ionotropic glutamate receptors (Tabor and Friedrich, 2008); therefore, treatment with kainic acid is expected to induce neural activity in the olfactory bulb. Adult fish were anesthetized with 4% tricaine (ethyl 3-aminobenzoate methanesulfonate solution; Sigma-Aldrich; Westerfield, 2000) and placed on a damp sponge. Kainic acid (Tocris Bioscience) was injected into the peritoneal cavity via a syringe (28G, Beckton Dickinson) at 10 mg per kg body mass in phosphate buffered saline (PBS). Control fish were injected with PBS only. The injected fish were placed in a recovery tank for 30 min and treated as above.

### Parapineal ablation

Parapineal ablation was performed on *Tg*(*foxd3*:*GFP*)^fkg17^, *Tg*(*foxd3*:*GFP*)^fkg17^; *Tg*(*-10lhx2a:GAP-EYFP*)^zf177^ or *Tg*(*foxd3*: *GFP*)^fkg17^; *Tg*(*-10lhx2a:SYP-GFP*)^zf186^ embryos at 2 or 3 dpf. Embryos were anesthetized with 0.04% tricaine (ethyl 3-aminobenzoate methanesulfonate solution; Sigma-Aldrich; Westerfield, 2000) and positioned dorsal side up in 1.2% low melting-temperature agarose under a 40 × water immersion lens. A scanning beam (885 nm excitation) from a multiphoton Ti:Sapphire laser (MaiTai HP, Spectra-Physics) mounted on a Leica SP5 confocal microscope was focused on the parapineal. Cell ablation was achieved by scanning with a 30–40% filter wheel (power) setting for 1 to 3 two-second passes. The extent of ablation was monitored with a 10% filter wheel setting after each scan pass until GFP was no longer detected. Successful ablation of the parapineal was re-evaluated by fluorescence microscopy prior to live imaging or fixation. In control larvae, cells in the brain were ablated on the opposite side of the pineal or adjacent to the parapineal.

### Imaging

Bright-field images were collected using an Axiocam HRc digital camera mounted on an Axioskop (Carl Zeiss). Fluorescent images were captured using a Zeiss Imager.Z1 microscope equipped with an AxioCam MRm digital camera or with a Leica SP5 confocal microscope. Images were pseudocolored and three-dimensional projections were generated using Imaris (Bitplane) or Axiovision (Carl Zeiss) software.

## Results

### *lhx2a*:*YFP* olfactory neurons project to a *fam84b*-expressing subregion of the right dorsal habenula

**Figure 1:**
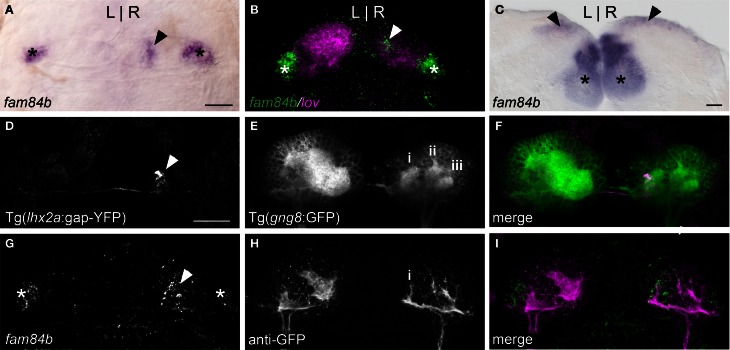
**A unique subnucleus in the right dorsal habenula of larval zebrafish. (A)** The *fam84b* gene is expressed in a medial domain in the right dorsal habenula (black arrowhead) and bilaterally in the ventral habenulae (asterisks in **A–C,G**). **(B)** The right-only *fam84b* domain (white arrowhead) is located within the *kctd 12.1* (lov)-expressing subnucleus, as demonstrated by fluorescent *in situ* hybridization. **(C)** *fam84b* is expressed bilaterally in the adult dorsal habenulae (black arrowheads). **(D–F)** Live imaging of *Tg*(*gng8*:*nfsB-CAAX-GFP*)^c375^ larva reveals that axons of *lhx2a:YFP* labeled olfactory mitral cells (white arrowhead in **D**) terminate at the medial neuropil (i in **E**) of the right habenula. More lateral neuropil clusters are indicated (ii,iii). **(G–I)** The medial neuropil cluster (i) visualized by anti-GFP immunofluorescence in *Tg*(*gng8*:*nfsB-CAAX-GFP*)^c375^ larva is closely associated with the *fam84b* expression domain (white arrowhead in **G**) revealed by fluorescent *in situ* hybridization. All larvae 4 dpf; scale bars = 50 μm.

**Figure 2:**
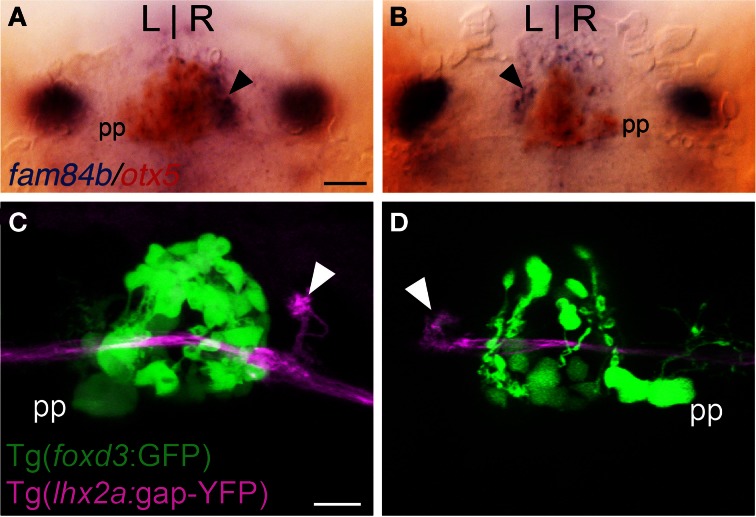
**Asymmetric olfactory input is contralateral to the parapineal. (A,B)** Larvae with the parapineal (pp) on the left of the pineal anlage exhibit *fam84b* expression (blue) in the right dorsal habenula (black arrow). Conversely, spontaneous L-R reversal of parapineal position corresponds with *fam84b* expression on the left. The pineal complex expresses the *otx5* gene (red), revealed by double label *in situ* hybridization methods. **(C,D)** Live imaging of *Tg*(*foxd3*:*GFP*)^fkg17^; *Tg*(*-10lhx2a:GAP-EYFP*)^zf177^ larvae. When the parapineal is on the left, *lhx2a:YFP* labeled terminals (white arrowhead) are found in the right dorsal habenula. With L-R reversal of epithalamic asymmetry, olfactory axons terminate in the left dorsal habenula. All larvae 4 dpf. Scale bars = 50 μm for **(A,B)** and 25 μm for **(C,D)**.

The dorsal habenular nuclei exhibit L-R differences in gene expression and in the extent of dense neuropil (Concha et al., 2000; Gamse et al., 2003, 2005), while the ventral nuclei are bilaterally symmetrical (Amo et al., 2010). The *fam84b* gene, which encodes a breast cancer membrane-associated protein (Adam et al., 2003) is transcribed bilaterally in the ventral habenulae, but is also expressed in an unusual pattern in only the right dorsal habenula (Figures 1A and 2A). The *fam84b* domain is within the lateral subnucleus of the right dorsal habenula (Figure 1B), defined by expression of the *kctd12.1* gene (Aizawa et al., 2005; Gamse et al., 2005). The medio-lateral designation of habenular subnuclei is based on their position in the adult brain, which differs from the larva due to extensive reorganization that occurs during development (Amo et al., 2009). Expression of *fam84b* is bilateral in the adult, but remains more extensive in the right dorsal habenula (Figure 1C).

Accumulations of neuropil differ between the left and right dorsal habenulae of larval zebrafish, as visualized by immunolabeling with antibodies against acetylated α-Tubulin (Concha et al., 2000; Taylor et al., 2011) or Synaptic Vesicle Protein 2 (SV2) (Hendricks and Jesuthasan, 2007; Miyasaka et al., 2009), and by labeling of membrane-tagged GFP in live *Tg*(*gng8*:*nfsB-CAAX-GFP*)^c375^ larvae. The left side has an expanded neuropil that extends the width of the dorsal habenula, while the right dorsal habenula has three more distinct, small clusters (Figure 1E). The most medial cluster corresponds to the site of innervation of *lhx2a*:YFP labeled axons from the olfactory bulb (Figures 1D–F) and overlaps with the *fam84b*-expressing cells (Figures 1G–I). The convergence of asymmetric pre-synaptic olfactory input, a discrete neuropil cluster and restricted *fam84b* expression suggests that the right dorsal habenula contains a functionally unique subnucleus.

### Directionality of olfactory innervation is determined by L-R asymmetry of the epithalamus

**Figure 3:**
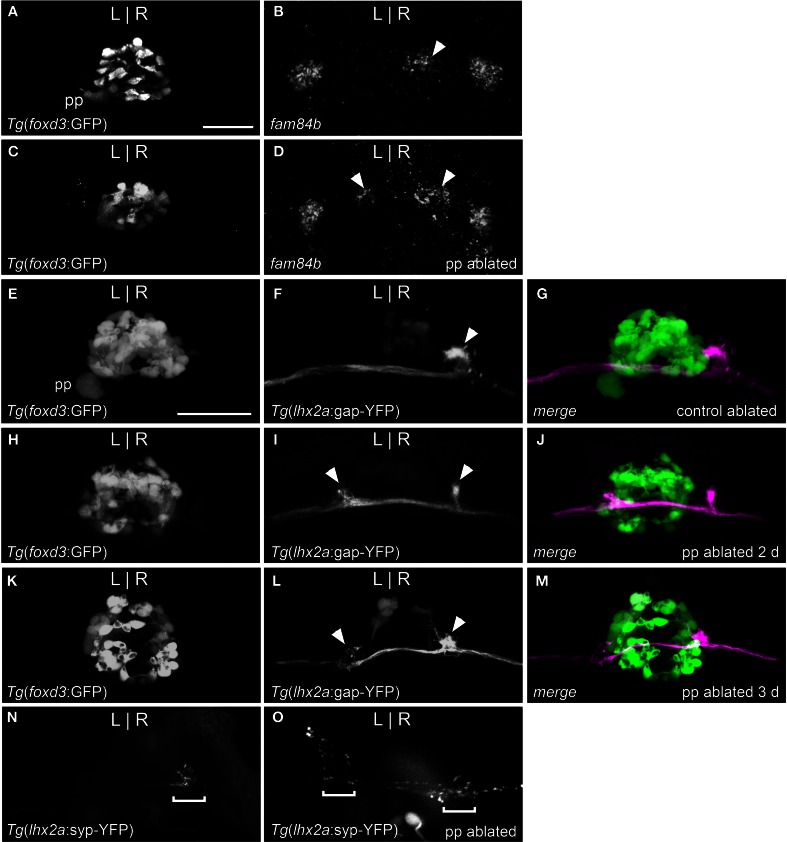
**Parapineal ablation results in symmetric OB-Ha pathway. (A)** Live imaging of *Tg*(*foxd3*:GFP)^fkg17^ larva, with parapineal (pp) situated to the left of the pineal. **(B)** Fluorescent *in situ* hybridization reveals *fam84b* transcripts in the right dorsal habenula (white arrow) and bilaterally in the ventral habenulae at 4 dpf. **(C)** Parapineal ablation at 2 dpf results in **(D)** bilateral *fam84b* domains (white arrowheads) in the dorsal habenulae. **(E–G)** *lhx2a:YFP* mitral cells (white arrowhead) innervate the right habenula of control-ablated larvae, but **(H)** in the absence of the parapineal, they terminate **(I,J)** at both habenulae (white arrowheads in **I**). **(K–M)** Parapineal ablation at 3 dpf also results in bilateral *lhx2a:YFP* projections (white arrowheads in **L**). **(N)** Region of synaptic vesicle density (demarcated by bracket) in the right dorsal habenula of *Tg*(*-10lhx2a:SYP-GFP*)^zf186^ larva is found **(O)** bilaterally following parapineal ablation. All images, except for **(B,D)** are of live larvae at 4 dpf, 5 dpf **(K–M)** and 6 dpf **(N,O)**. Scale bars = 40 μm.

Parapineal cells originate from the pineal anlage at 28 h post-fertilization (hpf) and migrate to the left side of the brain in >95% of embryos (Concha et al., 2003; Gamse et al., 2003). By 40 hpf, *kctd12.1* is strongly expressed in the presumptive left habenula and to a far lesser extent in the right. However, when the parapineal is found on the right side of the brain, either spontaneously (<5%) or following perturbation of Nodal-related signaling (Facchin et al., 2009), the asymmetric *kctd12.1* expression pattern is L-R reversed. Loss of the parapineal, either by laser-mediated cell ablation (Concha et al., 2003; Gamse et al., 2003) or the consequence of mutation (Snelson et al., 2008; Regan et al., 2009) results in symmetric development of the habenulae, with both the left and right nuclei exhibiting properties of the right habenula. Accordingly, when the parapineal is situated on the right side of the brain, *fam84b* is expressed in the left dorsal habenula (*n* = 3, Figure 2B) and, following ablation of the parapineal, *fam84b* domains are found bilaterally in both dorsal habenulae (control *n* = 4, Figures 3A,B; ablated *n* = 5, Figures 3C,D).

We examined whether altered directional asymmetry of the epithalamus also influences the connectivity of *lhx2a:YFP* labeled mitral cells. L-R reversal of parapineal position correlated with the olfactory bulb mitral cells innervating the left habenula rather than the right (Figures 2C,D). Compared to control ablated larvae with unilateral input to the right habenula (Figures 3E–G), lhx2a:YFP efferents innervate both dorsal habenulae in larvae with an ablated parapineal (*n* = 14, Figures 3H–J). These results indicate that L-R asymmetry of the epithalamic region directs olfactory connectivity. The bilateral projections likely form functional synapses, as evidenced by the localization of synaptic vesicles using *Tg*(*-10lhx2a:SYP-GFP*)^zf186^, in which a synaptophysin-YFP fusion protein is expressed under the control of the *lhx2a* cis-regulatory elements (Miyasaka et al., 2009). Clusters of synaptic vesicles were found in axon terminals at both dorsal habenulae (*n* = 3, Figures 3N,O). Ablation of the parapineal at a later developmental stage (77–80 hpf), following the establishment of the *lhx2a*:*YFP* olfactory-right habenula projection, also resulted in innervation of both dorsal habenulae (*n* = 13, Figures 3K–M). This reveals that the parapineal is required for the maintenance of dorsal habenular L-R identity, and that the OB-Ha pathway is highly plastic.

### Alarm pheromone does not stimulate the OB-Ha pathway

The dorsal habenulae have been implicated in modulating fear responses. Therefore, we examined whether substances known to provoke anxious behaviors in fish activate the asymmetric OB-Ha pathway in adult zebrafish. We used induction of *fos* expression as an indirect read-out of neural activity. This approach has been successfully applied in mammals to map the habenular response to a variety of stimuli (e.g., Kazi et al., 2004; Zhang et al., 2005; Paul et al., 2011) and the response to odorants in the olfactory bulb (e.g., Guthrie et al., 1993; Sallaz and Jourdan, 1993; Guthrie and Gall, 1995). We also examined expression of *egr1* but it was less robust than *fos* and was not detected in the olfactory bulb in response to chondroitin sulfate (*n* = 9, data not shown) or vehicle control (*n* = 5).

**Figure 4:**
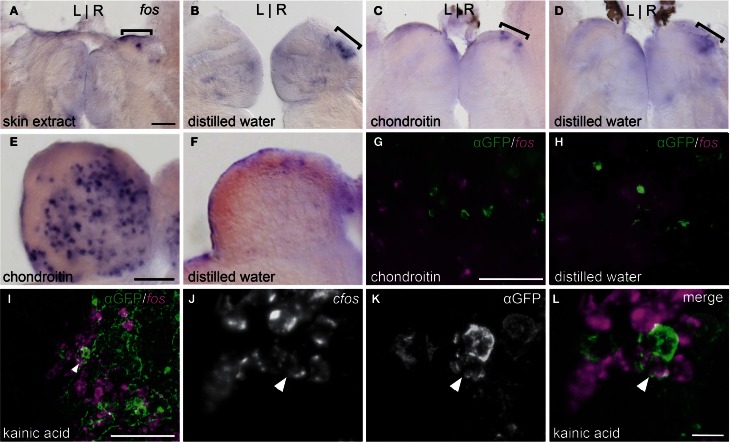
**Alarm substances do not stimulate the asymmetric OB-Ha pathway. (A,B)** Skin extract does not elicit higher *fos* expression (brackets) than controls in the dorsal habenula. **(C,D)** Chondroitin sulfate also does not activate *fos* expression above controls in the dorsal habenula. **(E,F)** Robust induction of *fos* expression in the olfactory bulb is observed following chondroitin sulfate exposure. **(G,H)** *fos* transcripts revealed by fluorescent *in situ* hybridization do not colocalize with immunolabeled *lhx2a:YFP* neurons in the olfactory bulb. **(I)** Kainic acid induces *fos* expression in a subset of *lhx2a:YFP* labeled olfactory neurons. **(J–L)** Higher magnification of **(I)**, with double labeled neuron indicated (white arrowhead). Coronal sections (50 μm) of adult brains. Scale bars = 40 μm for **(A–D), (E,F), (G,H),** and **(J–L)**, and 10 μm for **(I)**.

Exposure to skin extract (*n* = 11, Figure 4A) or purified shark chondroitin sulfate (*n* = 4, Figure 4C) did not elicit higher *fos* expression in the right dorsal habenula above control levels (*n* = 5, Figure 4B and *n* = 6, Figure 4D). In both treatment groups, a few *fos* positive neurons were found in the right habenula, but comparable expression was also observed in controls, suggesting that baseline *fos* induction is due to stimulus-unrelated activity caused by the experimental paradigm.

We also examined the response to chondroitin sulfate in the olfactory bulb. The olfactory bulb is comprised of several layers and, in zebrafish, mitral cell bodies reside within the glomerular and superficial inner cell layers while the interneuronal granule cells are found in a deeper position (Fuller et al., 2006). We observed that chondroitan sulfate treated individuals exhibited higher levels of *fos* expression in all regions (*n* = 4, Figure 4E) compared to controls (*n* = 6, Figure 4F). However, *fos* transcripts were never detected in *lhx2a:YFP* labeled mitral cells (chondroitin sulfate *n* = 8, control = 3, Figures 4G,H). In contrast, many olfactory bulb neurons including the *lhx2a*:YFP mitral cell subpopulation were activated with the glutamate receptor agonist kainic acid (Figures 4I–L). The centrally located fos positive cells are granule cells, whereas those found in the same layer or directly adjacent to *lhx2a*-expressing neurons are most likely mitral cells.

### Ecologically relevant odors stimulate non-*lhx2a:YFP* olfactory bulb neurons

All odorant treatments elicited a qualitatively higher *fos* response above negative control throughout the olfactory bulb region but not in *lhx2a:YFP* labeled mitral cells (data not shown, putrescine *n* = 4, cadaverine *n* = 3, trimethylamine *n* = 3, L-cysteine *n* = 4, fish flakes *n* = 5, conspecific extract *n* = 3, Tilapia bile *n* = 4).

## Discussion

The connection from primary sensory neurons in the olfactory epithelium to secondary mitral cells in the olfactory bulb that project directly to the right habenula provides a defined pathway to study the establishment and behavioral significance of L-R asymmetry in the vertebrate brain.

Previous studies demonstrated that the parapineal influences the identity of the adjacent dorsal habenula. When the parapineal is located to the right of the pineal, rather than at its typical left-sided position, the directionality of habenular L-R asymmetry is reversed. In the absence of the parapineal, the left and right dorsal habenular nuclei develop similarly, with gene expression patterns, neuroanatomical organization, and restricted efferent connections to the IPN characteristic of the right nucleus. Accordingly, we find that the parapineal also affects the formation of a subnucleus in the right habenula, defined by expression of *fam84b* and the absence of the olfactory cell adhesion molecule (OCAM; Miyasaka et al., 2009). This subnucleus is found within a region designated as the lateral subnucleus of the dorsal habenula (Aizawa et al., 2005). It also corresponds with the location of a coherent neuropil cluster and the site of axon terminals of *lhx2a:YFP* olfactory mitral cells. With a L-R reversal in parapineal position, this subnucleus is now found in the left dorsal habenula. Following ablation of the parapineal, *fam84b* expression is present bilaterally and both the left and right habenular nuclei are innervated by the *lhx2a:YFP* neuronal population. These findings indicate that L-R asymmetry of the epithalamic region is sufficient to direct telencephalic input. Thus, a small difference in one region of the brain (e.g., the parapineal) not only influences the development of neighboring structures (e.g., the dorsal habenulae), but also has farther-reaching consequences on pre-synaptic as well as post-synaptic neurons.

The molecular mechanisms that direct the asymmetric connection between *lhx2a:YFP* mitral cells and the right habenula have yet to be determined. One possibility is unilateral expression of an axon guidance molecule. The *neuropilin receptor 1a* (*nrp1a*) gene, for example, is expressed only by neurons in the left dorsal habenula (Kuan et al., 2007b). Its ligand, Sema3D, is produced in the midline rostral to the IPN and is important for guiding left habenula neurons to the dorsal region of the IPN (Kuan et al., 2007b). Innervation of the right dorsal habenula by *lhx2a:YFP* mitral cells is unaltered following depletion of Nrp1a (deCarvalho and Halpern, unpublished observations), suggesting that formation of the asymmetric OB-Ha pathway does not rely on repulsive Sema3D-Nrp1 signaling. An alternative hypothesis is that the *fam84b*-expressing cells provide an attractive signal to guide *lhx2a:YFP* axons to or stabilize their synapses at the right habenula. Fam84b (also known as breast cancer membrane protein 101 and NSE2) encodes a protein of unknown function that interacts with α1-Catenin and is localized to the membrane in regions of cell-cell contact (Adam et al., 2003). However, Fam84b itself is unlikely to be the guidance cue for innervating *lhx2a:YFP* axons as the appearance of *fam84b* transcripts follows rather than precedes olfactory axon outgrowth to the right habenula (deCarvalho and Halpern, unpublished observations). Although the cues that establish the asymmetric OB-Ha projection are unknown, localization of synaptic vesicles in axonal terminals at the right habenula strongly suggest that this is a functional connection. Confirmation of laterality in neuronal activity will come from transgenic approaches, such as selective optogenetic activation of the lhx2a-expressing subpopulation with expression of the calcium indicator GCaMP (e.g. Muto et al., 2011; Ahrens et al., 2012; Akerboom et al., 2012).

The identity of the *lhx2a:YFP*-expressing olfactory neurons does not lend much insight as to the odorant cues that stimulate this pathway. The *lhx2a:YFP* labeled primary sensory neurons are a subpopulation of crypt cells, one of three types of olfactory sensory neurons (OSNs) defined by their morphology in fish (Hansen and Zeiske, 1998). Crypt cells are relatively sparse compared to other OSNs (Hansen and Finger, 2000; Schmachtenberg, 2006) and, in zebrafish, appear to be nearly homogenous in their expression of the olfactory receptor gene, *ora4* (Oka et al., 2012). Ligands for the Ora4 receptor are unknown, although there is evidence in the trout that crypt cells are most sensitive to sexual pheromones (Vielma et al., 2008; Bazaes and Schmachtenberg, 2012). The restricted expression of *lhx2a* coupled with the fact that ~10% of OSNs are *ora4* negative, suggests that crypt cells are a diverse population and that the Ob-Ha pathway may sense a distinct odorant cue.

Because the *lhx2a:YFP* olfactory projections are located in an region of the OB responsive to the alarm pheromone component, chondroitin sulfate (Mathuru et al., 2012), and the dorsal habenulae have been implicated in the modulation of fear (Agetsuma et al., 2010; Lee et al., 2010), it was proposed that the asymmetric OB-Ha pathway in zebrafish mediates avoidance behavior elicited by alarm substances (Concha et al., 2012). To test this hypothesis, we measured *fos* expression in the olfactory bulb and dorsal habenulae following exposure to a variety of odorant cues. Induction of immediate early gene expression, such as *fos*, has been widely used as a read-out of olfactory stimulation in rodents (e.g., Guthrie et al., 1993; Sallaz and Jourdan, 1993; Guthrie and Gall, 1995; Okuyama et al., 2011; Bepari et al., 2012). In zebrafish, *fos* expression was an effective measure of neural activity upon light avoidance; (Lau et al., 2011) and in pharmacologically induced seizures (Baraban et al., 2005).

We found that a variety of complex and purified odorants produced robust *fos* expression in the olfactory bulb of adult zebrafish, supporting the validity of the assay. However, contrary to their proposed role, neither alarm pheromone nor chondroitin sulfate alone elicited *fos* activation in the *lhx2a:YFP* OB-Ha pathway. We did not detect colocalization of *fos* transcripts in *lhx2a:YFP* labeled mitral cells nor did we observe *fos* activation in the right habenula above control levels. We tested other odors known to be aversive or attractive cues, including polyamines that have been shown to elicit olfactory responses in fish (Friedrich and Korsching, 1998; Michel et al., 2003; Rolen et al., 2003) and may serve as feeding cues (Rolen et al., 2003). Additional attractive odors tested were food, conspecifics and bile, which is a social cue (Doving et al., 1980; Polkinghorne et al., 2001). We also examined the response to L-cysteine because zebrafish show strong avoidance to this odor (Vitebsky et al., 2005) and relatively high sensitivity compared to other amino acids (Michel and Lubomudrov, 1995). In all cases, *fos* expression was observed in cells in the OB but not in the *lhx2a:YFP* subpopulation. Thus, the *lhx2a:YFP* labeled pathway appears to be unresponsive to ecologically relevant odor cues known to elicit behavior in fish. Consistent with these findings, we also did not detect *fos* expression in the right dorsal habenula above baseline levels. Curiously, *fos* expression was frequently observed in the right habenula of controls as well as individuals exposed to odorants, suggesting that this activity was caused by some aspect of the experimental set-up or handling of the adult fish.

The most likely explanation for the absence of *fos* activation in *lhx2a-YFP* olfactory neurons is that a relevant odor was not tested. However, it is also possible that subcomponents of complex odorants such as fish flakes or conspecific extract masked or antagonized an activating cue (e.g., specific amino acids). Purified amino acids and specific components of sexual pheromones have been shown to elicit responses in the crypt cells of other fish species (Vielma et al., 2008; Bazaes and Schmachtenberg, 2012).

Alternatively, although the glutamate agonist kainic acid induced *fos* expression in *lhx2a:YFP* mitral cells, the *fos* response to odorants may be less robust in these neurons. Several functional mapping studies of the olfactory bulb in other species have demonstrated that mitral cells are competent to activate transcription of *fos* or other immediate early genes; however, expression is often reduced and less extensive than in the granule cell interneurons (e.g., Guthrie et al., 1993; Sallaz and Jourdan, 1993; Guthrie and Gall, 1995). Transcriptional activation is also known to differ in sensitivity among immediate early genes (Isogai et al., 2011; Bepari et al., 2012). For this reason, we examined expression of another immediate early gene, *egr1*, but in contrast to *fos, egr1* transcripts were not detected in the olfactory bulb following exposure to chondroitin sulfate. An alternative method may be required to assay neuronal activity in the asymmetrically projecting mitral cells, such as the use of genetically-encoded Ca^2+^ indicators.

It is also possible that the *lhx2a-YFP* labeled mitral cells consist of two distinct subpopulations, those that either express *fos* in response to kainic acid or do not. Neuroanatomical tracing of individual *lhx2a-YFP* mitral cells indicates that a fraction may terminate in the telencephalon (Miyasaka et al., 2009), whereas the majority projects to the right habenula. Whether these alternative projection patterns correspond to neurons with different sensitivities to *fos* activation remains to be determined.

Lateralization of olfactory systems is prevalent in invertebrates and appears to function either as a means to enhance odor discrimination or facilitate olfactory learning. For example, in *Caenorhabditis elegans*, bilaterally-paired sensory neurons express different olfactory receptors on the left and right side of the head (Wes and Bargmann, 2001). Asymmetry at the molecular level allows the worm to sense multiple odors using a limited number of OSNs. In the honeybee, individuals trained with their right antenna perform better on olfactory memory tasks than those trained with the left antenna (Letzkus et al., 2006). The right antenna seems to mediate short-term olfactory memory while the left is specialized for long-term memory retention (Rogers and Vallortigara, 2008). The higher number of sensilla on the right antennae compared to the left may account for lateralized olfaction in honeybees (Frasnelli et al., 2010). A recent study in *Drosophila* demonstrates that L-R differences may be quite subtle, in that differential neurotransmitter release between the ipsilateral and contralateral projections of single OSNs enables directional sensing of localized odorants (Gaudry et al., 2013).

Although the significance of asymmetry in zebrafish olfactory projections remains to be demonstrated, it may facilitate differential processing of stimuli as in invertebrate models. Several teleost species are known to exhibit L-R differences in their olfactory system. In flatfish such as the winter flounder, the upward facing right olfactory pathway from the olfactory epithelium through the telencephalon is significantly larger in volume (Prasada Rao and Finger, 1984) and L-R glomerular organization of the olfactory bulb is highly asymmetric in the turbot (Doldan et al., 2011). Studies of olfactory sensitivity in the Senegalese sole demonstrate tuning between the left and right olfactory epithelia to several different odorants (Velez et al., 2005, 2007).

The discovery of a specialized subnucleus in the right dorsal habenula of the zebrafish larva that is the site of olfactory innervation could provide important clues as to the downstream circuitry that mediates a lateralized response to environmental cues. The finding that directional asymmetry of the developing epithalamus guides the formation of this subnucleus reveals an unsuspected plasticity in olfactory connectivity and suggests that local L-R differences may have widespread consequences on neural pathways throughout the brain.

### Conflict of interest statement

The authors declare that the research was conducted in the absence of any commercial or financial relationships that could be construed as a potential conflict of interest.
